# A Simple Method for Noninvasive Quantification of Pressure Gradient Across the Pulmonary Valve

**DOI:** 10.1038/srep42745

**Published:** 2017-02-15

**Authors:** Xueying Zhou, Changyang Xing, Yang Feng, Yunyou Duan, Qiangsun Zheng, Zuojun Wang, Jie Liu, Tiesheng Cao, Lijun Yuan

**Affiliations:** 1 Department of Ultrasound Diagnostics, Tangdu Hospital, Fourth Military Medical University, Xi’an 710038, China; 2 Department of Cardiology, Tangdu Hospital, Fourth Military Medical University, Xi’an 710038, China

## Abstract

Pressure gradient across the pulmonary valve (PVPG) is an important hemodynamic variable used in the management of patients with cardiovascular and pulmonary disease. However, a reliable noninvasive method is unavailable. We hypothesized that a progressive Muller maneuver would elicit the pulmonary valve premature opening (PVPO) in diastole and that this event would be detectable by Doppler echocardiography. The intrathoracic pressure (ITP) decrease during this maneuver equals PVPG, which may be assessed with a custom airway pressure measurement device. A total of 102 subjects were enrolled in the study. At the earliest appearance of PVPO, the ITP decrease was recorded as the PVPG. PVPG was also simultaneously measured and compared by other two methods: right heart catheterization in 43 subjects, and routine Doppler echocardiography (pulmonary regurgitation jet) in the other 59 subjects. The results measured by different approaches were compared using the Bland-Altman analysis. PVPG assessed via PVPO showed strong agreement with PVPG measured by catheterization or routine Doppler echocardiography methods, with Lin concordance correlation coefficients of 0.91 and 0.70, respectively. In conclusion, PVPO provides a new noninvasive method of quantification of PVPG.

Diastolic pulmonary valve pressure gradient (PVPG) is a hemodynamic variable that contributes to the management of patients with cardiovascular and pulmonary conditions^1^,^2^,^3^. It can be used to evaluate the pulmonary artery diastolic pressure in patients with pulmonary hypertension and to predict heart failure hospitalization or cardiovascular death among ambulatory adults with coronary artery disease^1^,^4^, and has been proven to be an independent marker of cardiac dysfunction^5^. Also, diastolic PVPG is an important variable in deciding the left ventricular end-diastolic pressure when the pulmonary vascular resistance is normal^6^. The standard measurement of PVPG is via the cardiac cathether, which is invasive^7^.

Doppler echocardiography has significantly impacted clinical medicine by its ability to determine intracardiac hemodynamics noninvasively^8^,^9^,^10^,^11^. With the advent of the modified Bernoulli equation, Doppler echocardiographic techniques have allowed noninvasive estimation of PVPG, but under the limited condition of pulmonary regurgitation (PR)^1^,^12^.

Respiratory efforts, such as the Valsalva maneuver and the Muller maneuver, modulate intracardiac pressure via heart-lung interactions^13^,^14^,^15^. The Muller maneuver is performed by forcibly inspiring while the nose is held closed and the mouth sealed for about 10 seconds. It causes intrathoracic pressure (ITP) to decrease with no lung volume change^16^. In searching for alternative strategies for noninvasive intracardiac pressure measurement, we considered mechanical heart-lung interactions to approach this problem^17^,^18^,^19^.

As has been described before^20^, anatomically, despite of its complexity, the circulatory system can be regarded hydromechanically as two enclosed fluid systems which are connected sequentially (Fig. 1). Based on Pascal’s law, the ITP change during a gradual Muller maneuver may be transmitted without loss to all areas within the chest, including the fluid (blood) and soft tissues, which we assumed were incompressible. The fully-intrathoracic fluid, the dark portion in Fig. 1, is an enclosed fluid within the thoracic chamber; therefore, the ITP change may be transmitted to it. On the contrary, changes in ITP affect only the intrathoracic portion of the partially-intrathoracic fluid, the white portion in Fig. 1. With inspiration or the Muller maneuver, the right ventricular pressure decrease will be mostly compensated by venous return from the peripheral veins. Thus, the pressure in the right ventricle is not obviously decreased. In addition, the increased right ventricular stroke volume during ITP decrease rarely affects the pulmonary arterial diastolic pressure^21^. The unique anatomic arrangement of the two enclosed fluids establishes the conditions for an interesting hemodynamic phenomenon in the body. The change of ITP has very different influence on the two enclosed fluids. It equally decreases the pressure in the full-intrathoracic fluid that includes the pressure above the pulmonary valve (PV), but does not decrease the pressure below PV as this part belongs to the partially-intrathoracic fluid. That is the mechanism for the Muller maneuver to make PV to be opened in diastole. Normally, PV is closed during diastole since a positive pulmonary pressure is applied above the valve. To prematurely open PV in diastole, an ITP decrease has to be generated that is equal to PVPG. Since the ITP decrease equals the PVPG at the moment of pulmonary valve premature opening (PVPO) during the Muller maneuver (quantifiable by the intrapulmonary pressure measurement device)^18^, PVPG is obtained.

Based on the preceding analysis of ITP effects on hemodynamics, we found that a progressive Muller maneuver would elicit PVPO in diastole when the pulmonary artery pressure was at or below right ventricular pressure, and that this event would be detectable by Doppler echocardiography. The ITP decrease during this maneuver equals PVPG, which may be quantified with a custom airway pressure measurement device. Thus, PVPO provides a noninvasive method of quantification of pressure gradient across the pulmonary valve.

The purpose of this study was to test this new approach for noninvasive determination of PVPG by measuring the pressure at the time of PVPO using a device that permits quantification of ITP decrease during the Muller maneuver.

## Methods

The study complies with the Declaration of Helsinki and has been approved by the university Human Subjects Ethics Committee, and each subject provided an informed written consent.

### Subjects

One hundred and two subjects (52 women, 50 men; ages 14 to 73; mean age 39 years) were recruited for the present study, including 38 patients and 64 healthy subjects. Baseline characteristics were provided in Table 1. All subjects were instructed to perform standard Muller maneuver and those who failed were excluded from the study. All subjects were in sinus rhythm.

### Study groups

The PVPG of all 102 subjects was measured using the newly proposed technique – PVPO method. PVPG by invasive catheterization method (Catheterization method) was acquired in 43 of the 102 subjects for clinical reasons (including 5 healthy subjects with suspected peripheral vascular diseases). The patient selection and the data of catheterization were limited in this study as the university Human Subjects Ethics Committee does not permit using an invasive procedure on subjects only for research purpose. In this study, we might only have the subjects, the patients from cardiology division of our hospital, who clinically need pulmonary artery catheterization for their disease related hemodynamic data. Therefore, the pulmonary pressures of the patients included in this study were at random. PVPG by PR method was measured in the other 59 subjects during echocardiographic examination. The patients’ selection of this group was consecutive if their PRs were clear enough for the pressure gradient calculation.

### Devices and instruments

#### Intrapulmonary pressure measurement device

This device was purposely constructed to measure the intrapulmonary negative pressure generated by the Muller maneuver that has been previously described^18^. Similar customized devices have been widely used to quantify the intrathoracic pressure change caused by Muller or Valsalva maneuver^16^,^22^,^23^. As there are nearly no changes in lung volume during the Muller maneuver, the generated intrapulmonary negative pressure also represents the ITP decrease. The measurement device for the intrapulmonary pressure is an aneroid sphygmomanometer (Model XB-11 aneroid sphygmomanometer, Shanghai Medical Equipment Factory, Shanghai, China.) which is originally used for blood pressure measurement. We just used the manometer part and connected it with a mask and a pulse transducer which was used to display the action of the Muller maneuver.

Before a new aneroid manometer was used, it was calibrated routinely to avoid errors of pressure measurements. The method of the calibration is described in Supplementary Figure S1. The finger of the manometer was set at 100 mmHg before a measurement. During the Muller maneuver, the ITP decreased, applying a negative pressure to the manometer, so the finger would move counterclockwise from the 100 mmHg. The change of pressure from the manometer was recorded as ITP decrase.

#### Invasive measurements

A pulmonary artery catheter was used in the catheterization lab to measure the related hemodynamic data in those subjects who underwent invasive pressure measurement. A Philips M1567A single channel monitoring kit and transducer (Philips, Böblingen, Germany) was used for pressure measurements. The measurement with the PVPO was carried out right before the catheterization at the catheterization lab. They were not done simultaneously, but consecutively at the same bed without the patients’ moving.

#### Echocardiography

A Siemens Acuson Sequoia 512 ultrasonographic system (Mountain View, CA, USA) with 3V2c transducer (frequency = 2.5 to 4.0 MHz), and harmonic imaging was used for echocardiographic data collection. A thermo-sensitive transducer was used to record the natural respiration curve and a pulse transducer was refit to display the action of the Muller maneuver on the ultrasound instrument. The pulse transducer is originally a part of the ultrasound system used to measure pulse signal. It is used in this study to mark the start of Muller maneuver. A pressure change in the mask with the maneuver would be detected by the transducer and recorded as a deflection in the corresponding flat baseline.

For comparison of the new method with the PR method, the measurements with the new method were done right after the PR method at the echo lab. They were not done simultaneously, but consecutively at the same bed without the patients’ moving.

### Observations and measurements

#### Observation of natural PVPO

During natural respiration, the forward pulsed wave Doppler waveform of PV was recorded with the Acuson Sequoia 512 ultrasonographic system in all subjects. Attention was paid to natural PVPO, which occurs during spontaneous respiration.

#### End-diastolic PVPG measurements

PVPG was measured using the following three methods at end-inspiration:

##### PVPO method

This newly introduced noninvasive method was applied in each of the 102 subjects. Gradual Muller maneuvers were performed at end-expiration, with the inspiratory effort generated against a mask to prevent closure of the glottis, and ensuring the continuity of the manometer with the respiratory tract. The manometer was placed on one side of patient’s chest. It was noted that negative pressure may be generated in the mouth and produce a misleading pressure reading via sucking rather than inhaling. Subjects were instructed in the proper performance of the maneuver to avoid this problem.

At the beginning of a gradual Muller maneuver, additional negative pressure by the subject affects the thorax progressively, and the consequent reduction in ITP may be watched directly from the manometer. The rate of the pressure decrease can be controlled by the examinee, so the Muller maneuver was quantitatively controllable. At the same time, a pulsed wave Doppler echocardiographic technique was used to observe the PVPO by detecting the forward blood flow signal across the PV in diastole. The sample volume was placed above the PV orifice, and the blood flow velocity curve was continuously recorded. With the appearance of diastolic forward flow in the velocity profile, the examinee was instructed to remember the pressure reading at that moment from the manometer. The subject was asked to practice the Muller maneuver several times, and the data collection began once the pressure readings were consistently within 15% of one another. Three successive measurements were averaged and recorded as the PVPG obtained by the PVPO method. The subjects relaxed for at least 15 seconds between each maneuver.

##### Catheterization method

A conventional cardiac catheterization method was performed by means of pulmonary artery catheter in the catheterization lab immediately following the PVPO method. The invasive PVPG was acquired by subtracting the measured end-diastolic right ventricle pressure from the end-diastolic pulmonary artery pressure.

##### PR method

Continuous-wave Doppler echocardiography was employed to estimate the end-diastolic PVPG from the PR flow velocity tracing by means of the simplified Bernoulli equation: PVPG = 4V^2^, where V is the end-diastolic peak flow velocity of the PR jet. All data were measured three times and averaged for subsequent analyses.

### Statistical analysis

The agreement and correlation between the PVPO method and Catheterization method, and between the PVPO method and PR method, were assessed with Bland-Altman analysis, simple correlation and Lin concordance correlation coefficient. Inter- and intra-observer variability were performed in 13 subjects using the intra-class correlation coefficient and Bland-Altman analysis. The absolute difference between repeated measurements was reported.

## Results

### PVPO

**Figure 2:**
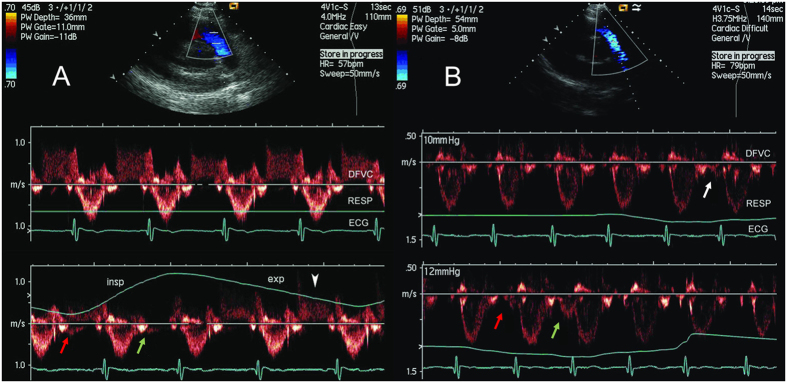
(**A**) Effects of respiration on the pulmonary regurgitation jet velocity and the pulmonary valve diastolic opening. The top panel shows a 2D echocardiogram in the parasternal short-axis view at the aortic valve level from a subject in the pulmonary regurgitation (PR) method group. The middle panel depicts the Doppler flow velocity curve (DFVC) of this healthy subject with mild PR. The Doppler flow velocity curve of the middle panel was recorded at end-expiration during breath holding. The peak PR velocity was slightly less than 1 m/sec, indicating that the pressure difference across pulmonary valve (PVPG) is close to 4 mmHg based on the modified Bernoulli equation. Note that the respiration curve is flat during the breath hold (RESP). The bottom panel showed that with the onset of inspiration (insp), the PR velocity decreased sharply and became erratic at the pulmonary valve orifice (red arrow, flow signals above and below the baseline). Subsequently, at end-inspiration, pulmonary arterial pressure was equal to or less than that in the right ventricle, and a discernable forward pulmonary arterial flow was detected (green arrow, PR nearly absent), indicating natural pulmonary valve diastolic opening. The PR velocity gradually increased with expiration (exp) and the waveform nearly normalized by end-expiration (arrowhead). (**B**) Exaggerated inspiratory effort leads to erroneous measurement of PVPG during the Muller maneuver. The top panel shows the same view as that in A, but from a subject in the Catheterization group. The middle panel shows the DFVC with the onset of a premature flow signal (arrow) at end-diastole during gradual Muller maneuver, indicating that at 10 mmHg of negative pressure, the pressure in the pulmonary artery is just below that of the right ventricle, the pulmonary valve opens prematurely, and forward flow occurs. The bottom panel demonstrates that with an exaggerated Muller maneuver, the premature flow was more prominent and was of greater velocity (green arrow) at 12 mmHg of negative pressure, resulting in an overestimation of PVPG.

Fourteen of the 43 subjects in the Catheterization method group (32%) and 20 of the 59 subjects in the PR method group (34%) had natural PVPO. All subjects demonstrated a PVPO in diastole with the Muller maneuver, across a variety of pressures. Figure 2 illustrated representiative results. Figure 2A showed the Doppler flow velocity curve of a representative subject from the PR method group with mild but adequate PR for PVPG measurement, and is also one of the 20 subjects who had natural PVPO. The effects of respiration on the PR velocity and the PVPO are clearly demonstrated. Note that the greatest PR velocity occurred at end-expiration. An intrathoracic negative pressure increase of 4 mmHg (from end-expiration) due to spontaneous inspiration might cancel the original PVPG of 4 mmHg, halt the PR of 1 m/sec and generate a very light, but discernable forward pulmonary arterial flow. The PVPG of this subject measured with the PVPO method was 5 mmHg, which was 1 mmHg different from the result using the Catheterization method. However, notice that the PVPO flow in Fig. 2A only appeared at early-diastole (at an ITP of −4 mmHg during a natural respiration), and to demonstrate it consistently at end-diastole, a more negative pressure (at least 1 mmHg) would likely be needed using the Muller maneuver. Note exaggerated inspiratory effort could lead to erroneous measurement of PVPG during the Muller maneuver (Fig. 2B lower panel).

### Agreement and Correlation

The mean values of PVPG were 13.2 mmHg in PVPO method and 11.5 mmHg in Catheterization method. Bland-Altman analysis showed that the mean difference in PVPG between PVPO + Catheterization methods was 1.7 mmHg and the range of the limits of agreement (LOA) was from −3.1 to 6.5 mmHg. There was excellent correlation between the PVPO and Catheterization amethods with a correlation coefficient (r) of 0.95 and Lin coefficient of 0.91 (95% CI 0.86–0.95).

**Figure 3:**
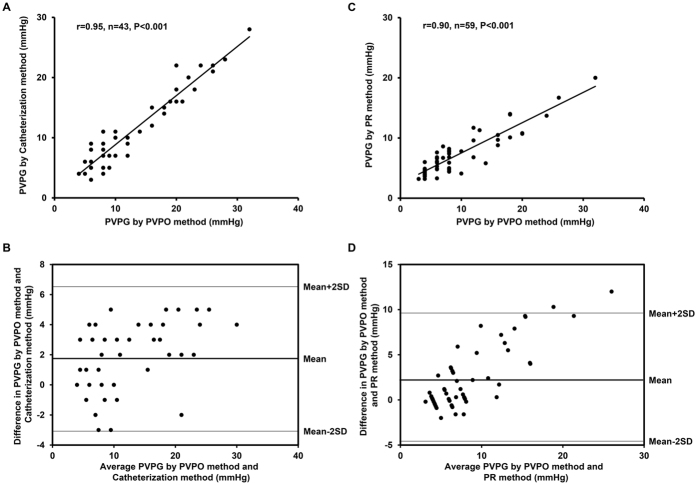
Correlation and agreement of measurements of pulmonary valve pressure gradient (PVPG) between pulmonary valve premature opening (PVPO) and Catheterization methods and between PVPO and pulmonary regurgitation (PR) methods.

The same analyses were done with the PVPO + PR method group. The mean values of PVPG were 9.4 mmHg in PVPO method and 7.2 mmHg in PR method, respectively. There were also good agreement and correlation between PVPO methods and PR methods (Fig. 3B,D). Bland-Altman analysis showed that the mean difference between the two methods was 2.2 mmHg and the range of LOA was from −4.9 to 9.3 mmHg. Statistical analyses revealed a correlation coefficient (r) of 0.90 and Lin coefficient of 0.70 (95% CI 0.61–0.77).

As shown in Fig. 3B, compared with the invasive method, the PVPO measures were prone to overestimate in subjects with higher PVPG. Thus, we performed a stratified analysis of agreement using 12 mmHg by invasive measurement as the cut-off. In both subgroups, PVPO measures overestimated the pressure gradient, which was more evident in the higher one. There was significant difference of the PVPG errors between subgroups (Low pressure gradient group vs. High pressure gradient group, 1.0 mmHg vs. 3.1 mmHg, P = 0.004).

### Intra- and inter-observer variability

**Figure 4:**
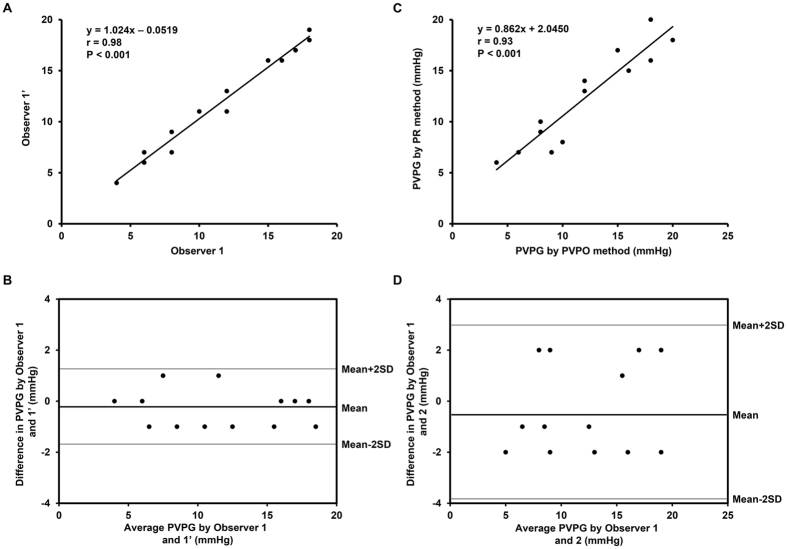
Intra- and inter-observer variability for pulmonary valve pressure difference (PVPG) measurements by the pulmonary valve premature opening method. Linear regression analysis showed excellent correlation and Bland-Altman analysis showed very good agreement between measurements taken by the same observer (**A**,**C**) and by two independent observers (**B**,**D**).

Linear regression analysis showed excellent correlation and Bland-Altman analysis showed very good agreement between measurements taken by the same observer (Fig. 4A,C) and by two independent observers (Fig. 4B,D) for the PVPG measurements by the PVPO method. The intra-class correlation coefficients of intra-observer variability and inter-observer variability were 0.98 (95% CI, 0.96–0.99) and 0.93 (95% CI, 0.79–0.98), respectively. The absolute difference between repeated measurements was 0.31 ± 0.75 mmHg and 0.31 ± 1.80 mmHg for the same observer and for two independent observers.

## Discussion

Doppler echocardiography, with its high spatial and superb temporal resolution, has revolutionized our clinical access to noninvasive assessment of cardiac physiology and pathophysiology^24^. In the present study, PVPO was elicited with the Muller maneuver and PVPG was acquired. PVPG measured by this new technique, the PVPO method, showed very good agreement with that measured by invasive right heart catheterization and the Doppler-derived PR method. This technique is simple, noninvasive and provides a quantitative tool to assess intracardiac hemodynamics beyond the modified Bernoulli equation. More importantly, it is a useful tool in the many patients who lack PR, or a suitable PR waveform for analysis of PVPG^4^,^12^.

There are several reports concerning noninvasive pulmonary artery diastolic pressure measurement by tricuspid regurgitation^25^,^26^. In the study of Lanzarini *et al*.^25^, they measured the systolic pressure gradient across the tricuspid valve as the pulmonary arterial diastolic pressure at the time of opening of the PV based on the assumption that the pulmonary arterial systolic pressure equalled pulmonary arterial diastolic pressure at that moment. Theoretically, that pressure gradient should be added with the estimated right atrial pressure, while numerically the pressure gradient itself was proven to correlate very well with the invasive pulmonary arterial diastolic pressure. Our result showed similar good correlation between intracardiac measurement and echocardiographic analysis as theirs, but better LOA (−3.1 ~ 6.5 mmHg, vs. −16 ~ 16 mmHg). Their wide range of LOA might attribute to the steep slope of the initial part of tricuspid regurgitation as stated in their critical reference that “The slope of the initial portion of the tricuspid regurgitant jet is relatively steep at the time of pulmonary valve opening…small errors can significantly alter the estimated gradient…”^26^. Our reduction of 10 mmHg random error in comparison with their method can be of important clinical significance.

Note that about 1/3 of the subjects had natural PVPO in diastole. Normally, the ITP variation over the respiratory cycle is about −4 mmHg (from end-expiration to end-inspiration). Based on the catheterization data, the normal right ventricular end-diastolic pressure is 4 mmHg and the pulmonary arterial diastolic pressure is 9 mmHg^27^, therefore, PVPG in normal subjects should be 9–4 = 5 mmHg. When the −4 mmHg respiratory ITP variation (average of −2 mmHg both on inspiration and expiration) is superimposed on the pulmonary arterial diastolic pressure, the PVPG varies approximately from 3 [5 + (−2)] to 7 [5-(−2)] mmHg with normal ITP variation generated by normal respiration. These pressure data suggest that PV should normally be closed but may open naturally during inspiration as seen in this study in some normal subjects with lower left ventricular diastolic pressure. The clinical meaning of the natural PVPO with natural respiration is that the PVPGs of these subjects are very small, or that the end-diastolic pressures of the left and right ventricles are very close and that if elevated right ventricular end-diastolic pressure can be excluded, the left ventricular end-diastolic pressure elevation and elevated pulmonary vascular resistance may also be excluded. About 1/3 of the subjects in this study had natural PVPO in diastole and we did not find evidence for their right ventricular end-diastolic elevation.

The major impact of the PVPG measurement is its role in noninvasively estimating the pulmonary artery diastolic pressure. By adding the noninvasively estimated right ventricular end-diastolic pressure to the end-diastolic PVPG, the pulmonary artery diastolic pressure may be calculated. The right ventricular end-diastolic pressure equals the right atrial diastolic pressure^21^, which can be obtained from the central venous pressure measurement by neck ultrasound^28^. Thus, the noninvasive PVPO method may be clinically useful as a routine assessment of pulmonary artery diastolic pressure.

It should be pointed out that there are several limitations in this study. First, PVPG may be overestimated if the ITP decrease generated by Muller maneuver is too large. To avoid this potential problem in this study, the Muller maneuver was quantitatively controlled in two ways. On one hand, the Muller maneuver was conducted slowly so that the pressure changed gradually. On the other hand, earliest onset of forward flow was detected with a highly sensitive technique - pulsed wave Doppler echocardiography. Nevertheless, the overestimation of PVPG still became more evident in the high pressure gradient subgroup. Although the mean bias of 3.1 mmHg in the high PVPG subgroup is clinically acceptable, the potential larger overestimation for subjects with higher pulmonary artery diastolic pressures, which are usually the cases in clinical practice referred for hemodynamic assessments, should be taken into consideration. Second, although we didn’t find adverse hemodynamic events when patients performed the Muller maneuver in our study cohort, we cannot rule out the possibility that the Muller maneuver may cause hemodynamic compromise for some patients in severe respiratory and unstable cardiac conditions. Meanwhile, this method is inapplicable to those who cannot perform adequate Muller maneuver. Last, our sample size is relatively small, so the generalizability needs further verification.

## Conclusions

This study provides a new noninvasive method of PVPG measurement using the PVPO method, which provides new insights into cardiac physiology, as well as a further understanding of the mechanism of respiration-driven hemodynamics.

## Additional Information

**How to cite this article**: Zhou, X. *et al*. A Simple Method for Noninvasive Quantification of Pressure Gradient Across the Pulmonary Valve. *Sci. Rep.* **7**, 42745; doi: 10.1038/srep42745 (2017).

**Publisher's note:** Springer Nature remains neutral with regard to jurisdictional claims in published maps and institutional affiliations.

## Supplementary Material

Supplementary Information

## Figures and Tables

**Figure 1:**
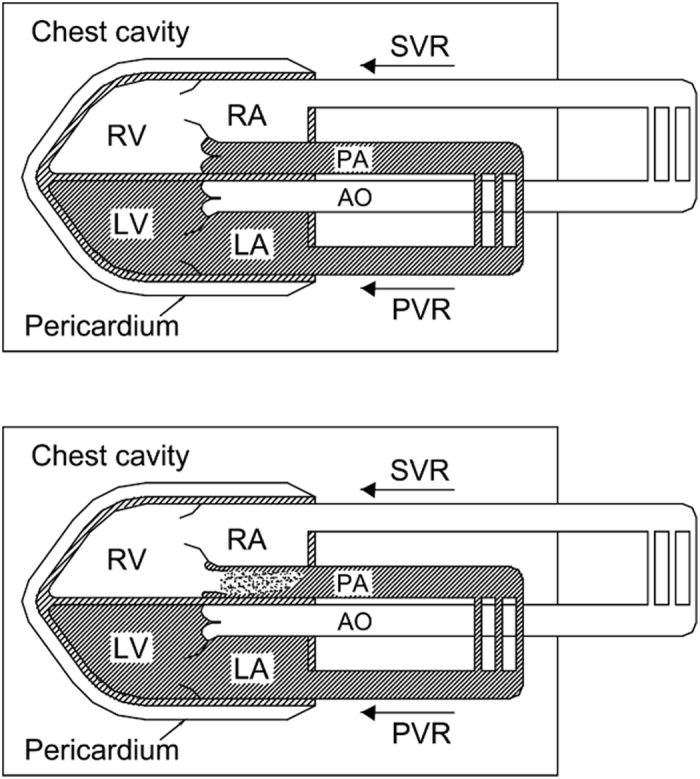
The mechanism of pulmonary valve diastolic opening (revised from ref. 20). Hydromechanically, the circulatory system may be considered as two enclosed fluid systems: fully-intrathoracic and partially-intrathoracic (the dark and the white portions, respectively). They are characteristically arranged in a closed chamber - the chest cavity - with negative pressure. With a gradual Muller maneuver or inspiration (see the bottom panel of Fig. 3A), the pressure in the fully-intrathoracic portion decreases equally with the decrease in intrathoracic pressure (ITP) (top panel), while the pressure decrease in the right ventricle (RV) will be mostly compensated for by the systemic venous return (SVR). With the progression of the maneuver, the pressure decrease in the pulmonary artery (PA) may drop below the pressure in the RV, and the pulmonary valve may open in diastole with a small diastolic forward flow (the bottom panel). This flow may be detected by a sensitive technique such as Doppler echocardiography. PVR, pulmonary venous return; LV, left ventricle; LA and RA, left and right atria; AO, aorta.

**Table 1:** Baseline characteristics.

Variable	Value
Age, y	39 ± 11
Sex, n, Male/Female	50/52
Weight, kg	63.1 ± 8.4
Height, cm	165.2 ± 6.6
BMI, kg/m^2^	23.1 ± 3.4
Systolic blood pressure, mmHg	122.8 ± 10.8
Diastolic blood pressure, mmHg	77.3 ± 7.5
Pulmonary artery systolic blood pressure*, mmHg	28.9 ± 7.7
Pulmonary artery diastolic blood pressure*, mmHg	13.1 ± 5.2
*Clinical conditions*	
Coronary heart disease, n	14
Idiopathic dilated cardiomyopathy, n	6
Hypertrophic cardiomyopathy, n	3
Primary hypertension, n	8
Suspected cardiac and vascular conditions, n	7
Healthy, n	64
*Comorbidities*	
Pulmonary hypertension, n	9
Heart failure, n	8
Chronic obstructive pulmonary disease, n	4

Data are given as mean ± SD unless otherwise mentioned. BMI indicates body mass index.

*Data from 43 subjects with invasive catheterization measurements.

## References

[b1] Rudski L. G. . Guidelines for the echocardiographic assessment of the right heart in adults: a report from the American Society of Echocardiography endorsed by the European Association of Echocardiography, a registered branch of the European Society of Cardiology, and the Canadian Society of Echocardiography. J Am Soc Echocardiogr 23, 685–713, quiz 786-688 (2010). 20620859 10.1016/j.echo.2010.05.010

[b2] Kenny D. . Percutaneous implantation of the Edwards SAPIEN transcatheter heart valve for conduit failure in the pulmonary position: early phase 1 results from an international multicenter clinical trial. J Am Coll Cardiol 58, 2248–2256 (2011). 22078433 10.1016/j.jacc.2011.07.040

[b3] Kohi M. P. . CMR assessment of right ventricular function in patients with combined pulmonary stenosis and insufficiency after correction of tetralogy of Fallot. Acta Radiol 54, 1132–1137 (2013). 23864059 10.1177/0284185113491565

[b4] Ristow B., Ali S., Ren X., Whooley M. A. & Schiller N. B. Elevated pulmonary artery pressure by Doppler echocardiography predicts hospitalization for heart failure and mortality in ambulatory stable coronary artery disease: the Heart and Soul Study. J Am Coll Cardiol 49, 43–49 (2007). 17207721 10.1016/j.jacc.2006.04.108 PMC2771184

[b5] Ristow B. . Pulmonary regurgitation end-diastolic gradient is a Doppler marker of cardiac status: data from the Heart and Soul Study. J Am Soc Echocardiogr 18, 885–891 (2005). 16153508 10.1016/j.echo.2005.06.004 PMC2776665

[b6] Shibata S. . Congestive heart failure with preserved ejection fraction is associated with severely impaired dynamic Starling mechanism. J Appl Physiol (1985) 110, 964–971 (2011). 21310890 10.1152/japplphysiol.00826.2010 PMC3075124

[b7] Doutreleau S. . Right Heart Hemodynamics in Pulmonary Hypertension- An Echocardiography and Catheterization Study. Circ J 80, 2019–2025 (2016). 27488283 10.1253/circj.CJ-16-0206

[b8] Anavekar N. S. & Oh J. K. Doppler echocardiography: a contemporary review. J Cardiol 54, 347–358 (2009). 19944309 10.1016/j.jjcc.2009.10.001

[b9] Ommen S. R. . Clinical utility of Doppler echocardiography and tissue Doppler imaging in the estimation of left ventricular filling pressures: A comparative simultaneous Doppler-catheterization study. Circulation 102, 1788–1794 (2000). 11023933 10.1161/01.cir.102.15.1788

[b10] Boyd J. H., Sirounis D., Maizel J. & Slama M. Echocardiography as a guide for fluid management. Crit Care 20, 274 (2016). 27592289 10.1186/s13054-016-1407-1 PMC5010858

[b11] Wright L. M., Dwyer N., Celermajer D., Kritharides L. & Marwick T. H. Follow-Up of Pulmonary Hypertension With Echocardiography. JACC Cardiovasc Imaging 9, 733–746 (2016). 27282440 10.1016/j.jcmg.2016.02.022

[b12] Lancellotti P. . Recommendations for the echocardiographic assessment of native valvular regurgitation: an executive summary from the European Association of Cardiovascular Imaging. Eur Heart J Cardiovasc Imaging 14, 611–644 (2013). 23733442 10.1093/ehjci/jet105

[b13] Ghazal S. N. Valsalva maneuver in echocardiography. J Echocardiogr (2016). 10.1007/s12574-016-0310-8 27515556

[b14] Iliceto S. . Effects of acute intrathoracic pressure changes on left ventricular geometry and filling. Am Heart J 116, 455–465 (1988). 3041789 10.1016/0002-8703(88)90618-7

[b15] Cassidy S. S. Heart-lung interactions in health and disease. Am J Med Sci 294, 451–461 (1987). 3321986 10.1097/00000441-198712000-00012

[b16] Wesley Reagan B., Helmcke F. & Kenneth Kerut E. Commonly used respiratory and pharmacologic interventions in the echocardiography laboratory. Echocardiography 22, 455–460 (2005). 15901303 10.1111/j.1540-8175.2005.40095.x

[b17] Yuan L. . Noninvasive assessment of influence of resistant respiration on blood flow velocities across the cardiac valves in humans–a quantification study by echocardiography. Echocardiography 21, 391–398 (2004). 15209717 10.1111/j.0742-2822.2004.03086.x

[b18] Wang Z. . Simultaneous beat-by-beat investigation of the effects of the Valsalva maneuver on left and right ventricular filling and the possible mechanism. PLoS One 8, e53917 (2013). 23342040 10.1371/journal.pone.0053917 PMC3544743

[b19] Neumann P. . Hemodynamic effects of spontaneous breathing in the post-operative period. Acta Anaesthesiol Scand 49, 1443–1448 (2005). 16223387 10.1111/j.1399-6576.2005.00868.x

[b20] Xing C. Y. . Mechanism study of pulsus paradoxus using mechanical models. PLoS One 8, e57512 (2013). 23469010 10.1371/journal.pone.0057512 PMC3585346

[b21] Godje O. . Central venous pressure, pulmonary capillary wedge pressure and intrathoracic blood volumes as preload indicators in cardiac surgery patients. Eur J Cardiothorac Surg 13, 533–539, discussion, 539–540 (1998). 9663534 10.1016/s1010-7940(98)00063-3

[b22] Scharf S. M., Bianco J. A., Tow D. E. & Brown R. The effects of large negative intrathoracic pressure on left ventricular function in patients with coronary artery disease. Circulation 63, 871–875 (1981). 7471344 10.1161/01.cir.63.4.871

[b23] Stowhas A. C. . The effect of simulated obstructive apnea and hypopnea on aortic diameter and BP. Chest 140, 675–680 (2011). 21393398 10.1378/chest.10-2799

[b24] Lester S. J. . Unlocking the mysteries of diastolic function: deciphering the Rosetta Stone 10 years later. J Am Coll Cardiol 51, 679–689 (2008). 18279730 10.1016/j.jacc.2007.09.061

[b25] Lanzarini L., Fontana A., Lucca E., Campana C. & Klersy C. Noninvasive estimation of both systolic and diastolic pulmonary artery pressure from Doppler analysis of tricuspid regurgitant velocity spectrum in patients with chronic heart failure. Am Heart J 144, 1087–1094 (2002). 12486435 10.1067/mhj.2002.126350

[b26] Stephen B., Dalal P., Berger M., Schweitzer P. & Hecht S. Noninvasive estimation of pulmonary artery diastolic pressure in patients with tricuspid regurgitation by Doppler echocardiography. Chest 116, 73–77 (1999). 10424506 10.1378/chest.116.1.73

[b27] Mann D. L., Zipes D. P., Libby P., Bonow R. O. & Braunwald E. In Braunwald’s heart disease: a textbook of cardiovascular medicine 364–391 (Elsevier/Saunders, 2015).

[b28] Xing C. Y. . New method for noninvasive quantification of central venous pressure by ultrasound. Circ Cardiovasc Imaging 8 (2015). 10.1161/CIRCIMAGING.114.003085 25904575

