# Supplementary material for: A Simple Method for Noninvasive Quantification of Pressure Gradient Across the Pulmonary Valve
Source: Sci Rep. 2017 Feb 15;7:42745. doi: 10.1038/srep42745 (PMC5309885; doi:10.1038/srep42745)
Supplement: Supplementary Information [file srep42745-s1.pdf]

Title: A Simple Method for Noninvasive Quantification of Pressure Gradient across the Pulmonary Valve

Xueying Zhou[1,+], Changyang Xing[1,+], Yang Feng[1,+], Yunyou Duan[1], Qiangsun Zheng[2], Zuojun Wang[1], Jie Liu[1], Tiesheng Cao[1,*] & Lijun Yuan[1,*]

[1]Department of Ultrasound Diagnostics, Tangdu Hospital, Fourth Military Medical University, Xi'an 710038, China

[2]Department of Cardiology, Tangdu Hospital, Fourth Military Medical University, Xi'an 710038, China

*** Correspondence to:**

Tiesheng Cao (email: caotsxcy@hotmail.com) or Lijun Yuan (yuanlj@fmmu.edu.cn)

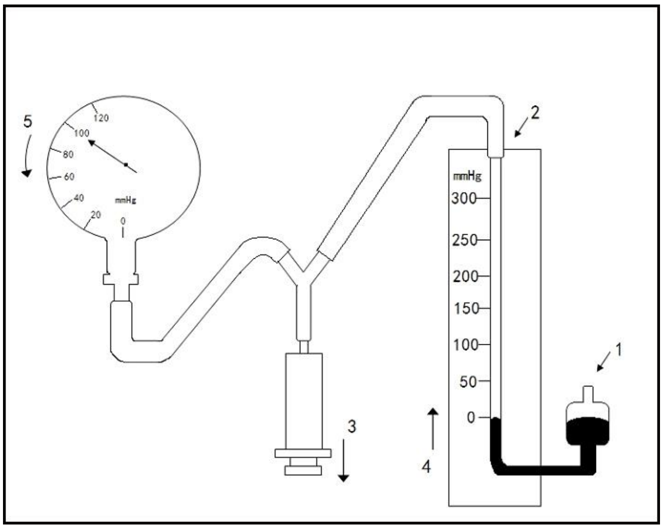

Figure S1. Sketch map of the setup for the aneroid manometer negative pressure calibration. The aneroid manometer (left on the sketch) is detached from an aneroid blood pressure manometer. To measure the negative pressure generated with Muller maneuver, we set the finger of the manometer at 100mmHg. When a positive pressure is applied on the aneroid manometer, the finger will move clockwise from the 100mmHg which actually means from 0mmHg and when the negative pressure is applied, the finger will move counterclockwise from the 100mmHg. We used a mercury manometer (right on the sketch) as a standard for calibration. It is a classical mercury-type sphygmomanometer for blood pressure measurement. The mercury manometer was used and the other parts were taken away at the point of arrow No.1. The end at arrow No.1 was opened to the atmospheric pressure. The cap (arrow No. 2) of the glass tube of the mercury column was also taken away for the connection of the rubber tubing with the other parts of the calibration system. The syringe (middle on the sketch) and the aneroid manometer (left on the sketch) were connected with the mercury manometer. When the handhold of the syringe moves down (arrow No. 3), it generates a negative pressure in the tubes. The mercury column moves up (arrow No. 4) indicates the standard negative pressure in the system, while the finger of the aneroid manometer goes counterclockwise to also indicate the negative pressure.
